# *PRRT2* mutations lead to neuronal dysfunction and neurodevelopmental defects

**DOI:** 10.18632/oncotarget.9258

**Published:** 2016-05-09

**Authors:** Yo-Tsen Liu, Fang-Shin Nian, Wan-Ju Chou, Chin-Yin Tai, Shang-Yeong Kwan, Chien Chen, Pei-Wen Kuo, Po-Hsi Lin, Chin-Yi Chen, Chia-Wei Huang, Yi-Chung Lee, Bing-Wen Soong, Jin-Wu Tsai

**Affiliations:** ^1^ Department of Neurology, Neurological Institute, Taipei Veterans General Hospital, Taipei, Taiwan; ^2^ Department of Neurology, National Yang-Ming University School of Medicine, Taipei, Taiwan; ^3^ Program in Molecular Medicine, National Yang-Ming University and Academia Sinica, Taipei, Taiwan; ^4^ Institute of Brain Science, National Yang-Ming University, Taipei, Taiwan; ^5^ Istitute of Pharmaceutics, Development Center for Biotechnology, New Taipei City, Taiwan; ^6^ Graduate Institute of Life Sciences, National Defense Medical Center, Taipei, Taiwan; ^7^ Brain Research Center, National Yang-Ming University, Taipei, Taiwan; ^8^ Biophotonics and Molecular Imaging Research Center, National Yang-Ming University, Taipei, Taiwan

**Keywords:** PRRT2, neuronal migration, synaptic development, paroxysmal kinesigenic dyskinesia (PKD), Taiwan, Pathology Section

## Abstract

Mutations in the proline-rich transmembrane protein 2 (*PRRT2*) gene cause a wide spectrum of neurological diseases, ranging from paroxysmal kinesigenic dyskinesia (PKD) to mental retardation and epilepsy. Previously, seven PKD-related *PRRT2* heterozygous mutations were identified in the Taiwanese population: P91QfsX, E199X, S202HfsX, R217PfsX, R217EfsX, R240X and R308C. This study aimed to investigate the disease-causing mechanisms of these *PRRT2* mutations. We first documented that Prrt2 was localized at the pre- and post-synaptic membranes with a close spatial association with SNAP25 by synaptic membrane fractionation and immunostaining of the rat neurons. Our results then revealed that the six truncating Prrt2 mutants were accumulated in the cytoplasm and thus failed to target to the cell membrane; the R308C missense mutant had significantly reduced protein expression, suggesting loss-of function effects generated by these mutations. Using *in utero* electroporation of shRNA into cortical neurons, we further found that knocking down Prrt2 expression *in vivo* resulted in a delay in neuronal migration during embryonic development and a marked decrease in synaptic density after birth. These pathologic effects and novel disease-causing mechanisms may contribute to the severe clinical symptoms in *PRRT2*–related diseases.

## INTRODUCTION

**Figure 1:**
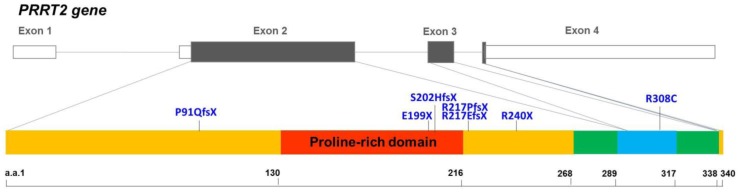
Genomic organization of the human PRRT2 gene and the distribution of the PRRT2 mutations identified in Taiwanese PKD patients The genomic organization of human *PRRT2* gene and the distribution of the seven *PRRT2* mutations identified in Taiwanese PKD patients are illustrated in the upper panel. The predicted domains of the PRRT2 protein are shown in the lower panel. Six of the *PRRT2* truncating mutations are localized in the predicted extracellular domain (amino acids 1-268), and five are clustered within or around the proline-rich domain (the red region).

Mutations in the proline-rich transmembrane protein 2 gene (*PRRT2*, NM_145239.2) have been identified to be the causes of many neurological diseases. Heterozygous *PRRT2* mutations cause paroxysmal kinesigenic dyskinesia (PKD), benign familial infantile epilepsy (BFIE), infantile convulsions and choreoathetosis (ICCA), hemiplegic migraine (HM), episodic ataxia (EA), paroxysmal torticollis, or a combination of them [1–14]. Although rarely identified, patients with biallelic or homozygous *PRRT2* mutations presented complex forms of PKD in combination with intellectual disability or cerebellar atrophy [10, 15, 16]. Previously, our group identified seven different PKD-related *PRRT2* mutations in the Taiwanese population: p.P91Qfs*24 (P91QfsX), p.E199X (E199X), p.S202Hfs*16 (S202HfsX), p.R217Pfs*8 (R217PfsX), p.R217Efs*12 (R217EfsX), p.R240X (R240X) and p.R308C (R308C) [11, 17]. Six of these mutations result in premature truncation before the first predicted transmembrane domain. The only missense mutation, R308C, is located in the predicted cytoplasmic domain (Figure 1). There were a few *in vitro* studies suggesting that PRRT2 may interact with SNAP25 (the synaptosomal-associated protein, 25kD), a critical molecule in neurotransmitter release [18, 19]. However, it is still not fully understood how mutated PRRT2 causes pathological effects on the nervous system *in vivo*.

Here, we generated a polyclonal antibody against Prrt2 which helped document that Prrt2 was localized at both the pre- and post-synaptic membranes with a close spatial association with SNAP25. We then revealed that abnormal intracellular trafficking and decreased expression of the aforementioned seven *PRRT2* mutations could lead to its functional loss. Furthermore, by *in utero* electroporation of Prrt2 shRNA into mouse embryos, we showed that Prrt2 knockdown in cortical neurons caused a delay in neuronal migration and defects in synaptic development. These pathological effects may contribute to the severe clinical syndromes in patients with homozygous or biallelic *PRRT2* mutations.

## RESULTS

### Production of a highly specific Prrt2 polyclonal antibody

**Figure 2:**
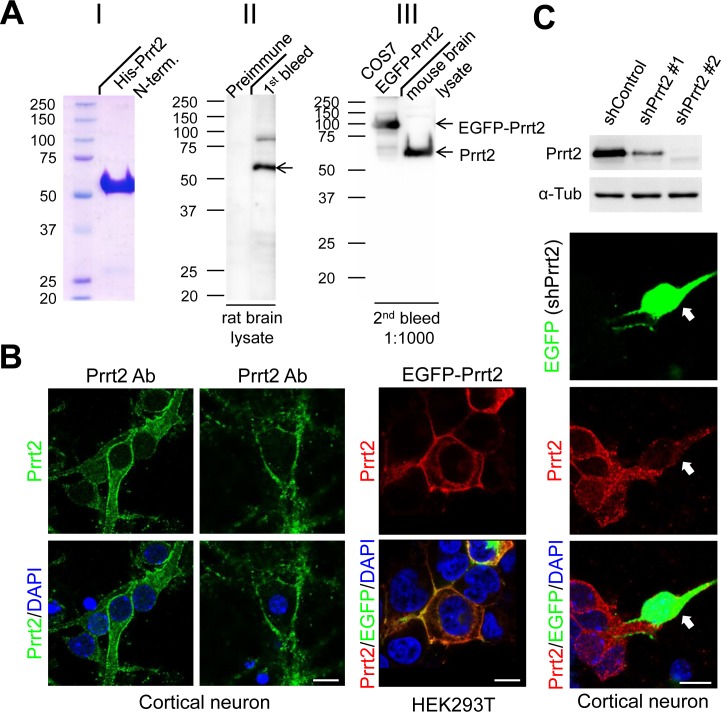
Prrt2 protein recognized by a specific polyclonal antibody **A.** A polyclonal antibody against amino acids 1-268 of the bacterially-expressed Prrt2 protein (I) was generated. Prrt2 protein from adult rat brain lysates was detected using the first bleed of Prrt2 anti-serum obtained from a rabbit (II, right lane). Preimmune serum drawn from the rabbit before antigen injection was the control (II, left lane). The antibody also recognized Prrt2 in mouse brain lysates and EGFP-tagged Prrt2 transfected into COS7 cells (III). **B.** Subcellular localization of Prrt2 protein by immunoflourescence staining with the polyclonal antibody. Endogenous Prrt2 protein (green) localized predominantly to cell membranes in cultured cortical neurons 3 and 16 days *in vitro* (DIV) (left panel). The distribution of EGFP-tagged Prrt2 also localized to the cell membrane in HEK293T cells transfected with EGFP-Prrt2, as revealed by the EGFP signal (green) and Prrt2 immuostaining (red). The distribution of Prrt2 based on EGFP signal and antibody staining was highly concordant and resembled the pattern of endogenous Prrt2 in cultured neurons. All cells were counterstained with DAPI staining (blue) to show the nuclei. Scale bar = 10 μm. **C.** Prrt2 protein level revealed by immunostaining with polyclonal antibody in cultured neurons transfected with Prrt2 shRNA. Upper panel: Prrt2 expression was knocked down by cotransfecting short hairpin RNA (shRNA) with Prrt2 into cultured cells. Two shRNA plasmids targeting different Prrt2 mRNA regions showed 65% (shPrrt2 #1) and 98% (shPrrt2 #2) knockdown efficiency 48 hours after transfection. Lower panel: Immunostaining in cultured neurons shows that endogenous Prrt2 signal (red) was significantly reduced in shPrrt2-transfected neurons (green, arrows) compared with the surrounding untransfected cells. All cells were counterstained with DAPI staining (blue) to show the nuclei. Scale bar = 10 μm.

To detect endogenous Prrt2, we first generated a polyclonal antibody against the extracellular domain of the Prrt2 protein and tested its specificity in rat and mouse brain lysates, as well as in COS-7 cells transfected with EGFP-Prrt2 construct (Figure 2A). The antibody showed high specificity for Prrt2, revealing a single major band (~ 65 kD) with few minor bands in each system using Western blotting. By using the antibody, endogenous Prrt2 was shown to localize at the cell membrane of mouse cortical neurons cultured for 3 or 16 days *in vitro* (DIV) (Figure 2B, left and middle panels). We also examined the distribution of EGFP-Prrt2 in transfected cultured HEK293T cells (Figure 2B, right panel). The fluorescence signals from EGFP and Prrt2 antibody predominantly co-localized at the cell membrane, consistent with previous observations using other protein tags [1, 20]. To further test the specificity of the antibody, we test whether knocking down Prrt2 expression in cells by RNA interference (RNAi) will eliminate antibody reactivity (Figure 2C). Two short hairpin RNA (shRNA) plasmids targeting different Prrt2 mRNA regions were used and showed 65% (shPrrt2 #1) and 98% (shPrrt2 #2) knockdown efficiencies 48 hours after transfection. We then transfected these shRNA constructs to cultured cortical neurons and found that Prrt2 signal was significantly lower than that in the surrounding non-transfected cells, indicating that the polyclonal antibody is highly specific.

### Close co-localization of Prrt2 and SNAP25 on the pre- and post-synaptic membranes

**Figure 3:**
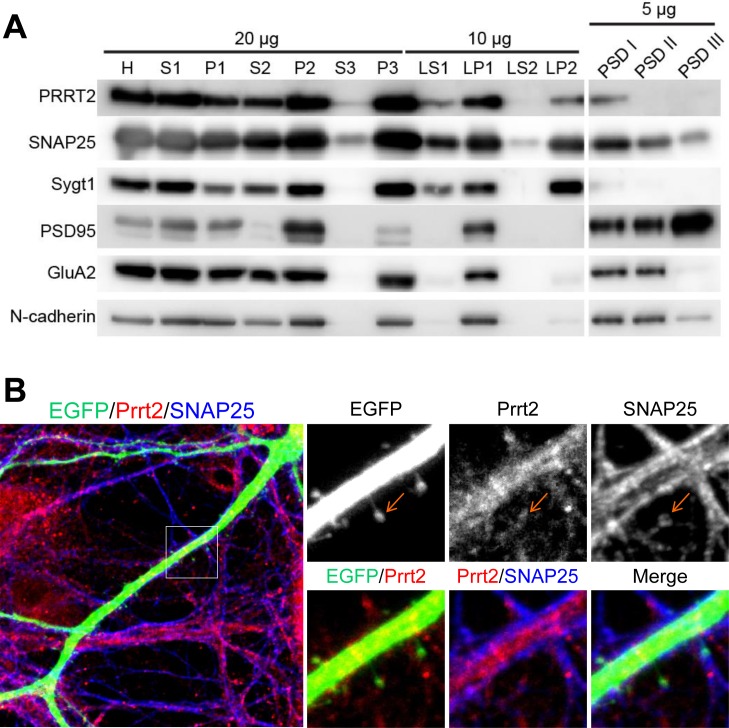
Localization of Prrt2 at the pre- and post-synaptic membranes **A.** Membrane fractionation assay and post-synaptic density (PSD) preparation of the protein from an adult rat brain. Prrt2 and SNAP25 were present and co-fractionated in both pre- and post-synaptic compartments. PSD95 and GluA1 are PSD proteins that were enriched in PSD fractions. Synaptotagmin I (Sygt1)-a presynaptic vesicle protein was present in the LP2 fraction, but absent from all PSD fractions. PSD95, GluA2 and N-cadherin are post-synaptic membrane proteins that were absent from the LP2 fraction. H = homogenate, S1 = low-speed supernatant, P1 = nuclear pellet and debris, S2 = cytosol plus light membrane, P2 = crude synaptosomal fraction, S3 = cytosol, P3 = light membranes, LS1 = synaptosomal cytosolic fraction, LP1 = synaptosomal membrane fraction, LS2 = luminal fraction of synaptic vesicles, LP2 = synaptic vesicle membrane fraction. PSDI, II and III represent the detergent extractability of PSD resident proteins. PSD1 represents Single Triton X-100 extraction. PSDII and PSDIII were obtained in the second round of Triton extraction and Sarkosyl extraction of PSDI, respectively. **B.** Immunostaining of Prrt2 and SNAP25 in GFP-expressing cultured hippocampal neurons (DIV 17). A dendritic segment with multiple spines (specialized protrusive structures) is shown in the large image. The amplified images highlight the localization of Prrt2 (red) and SNAP25 (blue), which co-localized at the tip of a spine (orange arrow) where a synapse was forming.

Synaptic membrane fractionation and postsynaptic density (PSD) preparation from adult rat brain lysates were carried out with several pre- and post-synaptic markers to confirm if Prrt2 is present on synapses (Figure 3A). Prrt2 was enriched in the synaptic membrane fraction (LP1), but was also detected in synaptic vesicle (LP2) and postsynaptic density (PSDI) fractions, indicating the presence of Prrt2 at both pre- and post-synaptic membranes. To further characterize the subcellular localization of Prrt2, we visualized the protein along dendritic spines in mature cultured hippocampal neurons of adult rats. Prrt2 was visible at the tips of these spines where the majority of excitatory synapses are formed [21, 22], with a close spatial association with SNAP25 (Figure 3B).

### Failure of membrane targeting in truncated Prrt2 mutants

**Figure 4:**
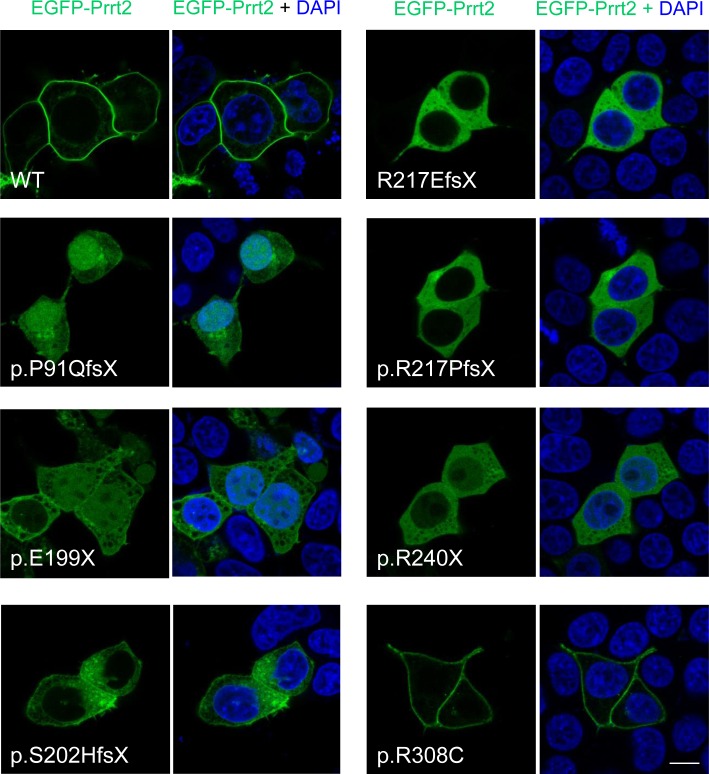
Subcellular localization of PKD-related PRRT2 mutations in cultured HEK293T cells The six prematurely truncating Prrt2 mutants (P91QfsX, E199X, S202HfsX, R217EfsX, E217PfsX and R240X) were retained in the cytoplasm and failed to be properly transported to the membrane. Some faint signal from the p.P91QfsX, p.E199X and p.S202HfsX Prrt2 mutant proteins could be found in the nucleus. The missense R308C mutant protein had a membrane localization similar to the pattern for WT-Prrt2. Scale bar = 10 μm.

The subcellular localization of the seven PKD-related mutant Prrt2 was then explored in cultured cells (Figure 4). HEK293T cells were transfected with EGFP-Prrt2 or its mutants and imaged with confocal microscopy. Wild type (WT) Prrt2 protein predominantly localized to the plasma membrane, consistent with the pattern observed for endogenous Prrt2 in cultured mouse neurons. In contrast, the six truncated Prrt2 mutants were mostly retained in the cytoplasm, with some mutant proteins (P91QfsX, E199X and R240X) found in the nucleus, indicating abnormal intracellular trafficking of the mutant proteins. Of note, the R308C mutant was expressed on the plasma membrane in a pattern indistinguishable from WT-Prrt2.

### Reduced Prrt2 protein level caused by the R308C mutation

**Figure 5:**
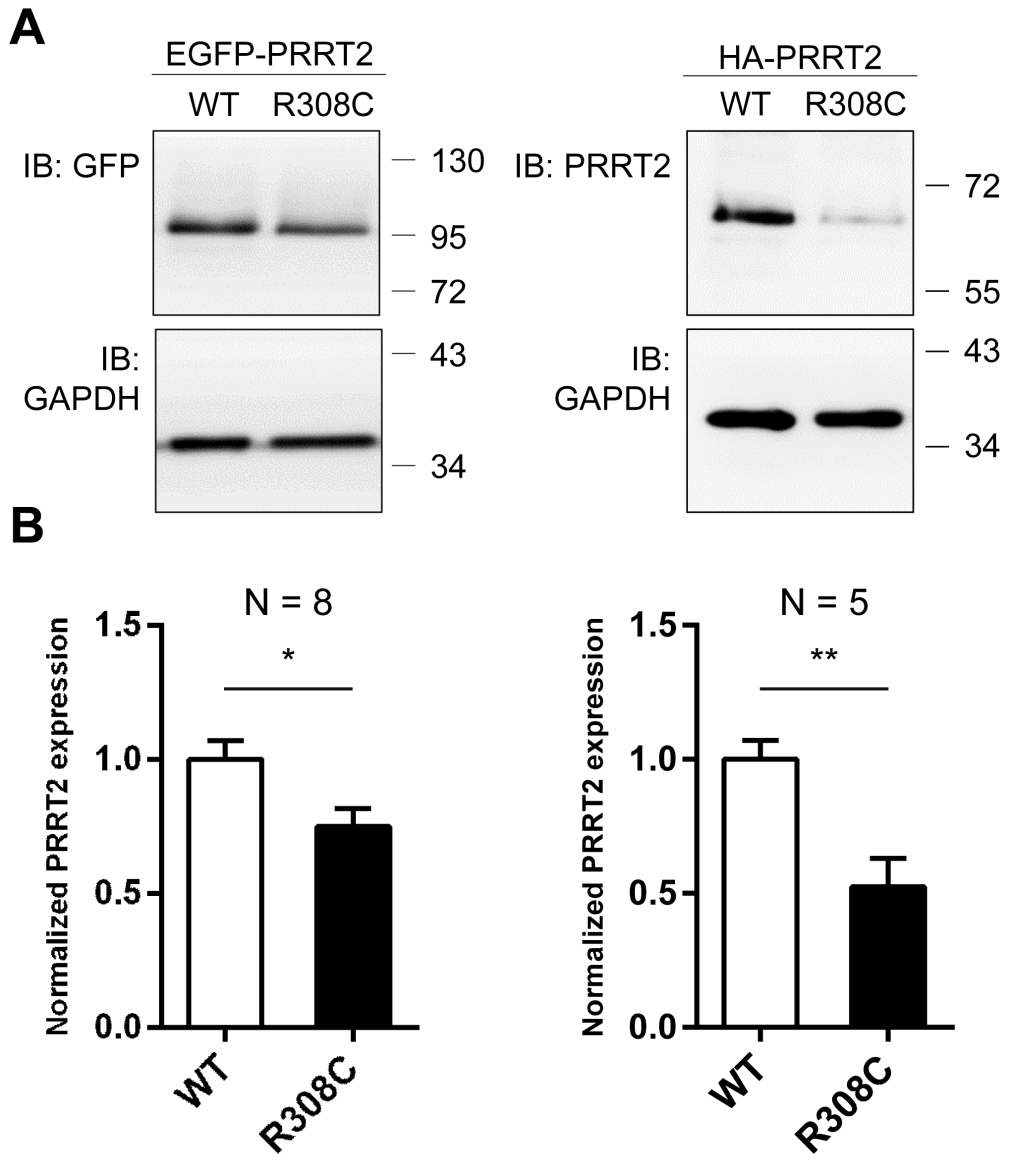
Reduced expression level of the R308C PRRT2 mutant **A.** GFP- and HA-tagged WT-Prrt2 and the R308C mutant were transiently transfected into the HEK293T cell line. Prrt2 expression levels were determined using Western blotting and were normalized to that of WT-Prrt2. GAPDH was used as an internal control. **B.** A significant reduction of Prrt2 levels was observed for the R308C mutant (*: *p* < 0.05, **: *p* < 0.01). Error bars indicate SEM. Statistics were performed by a two-tailed, unpaired Student's t-test.

Although the R308C mutant was found to localize at the plasma membrane as WT, it is possible that insufficient level of mutant protein causes dysfunction of PRRT2. To address this possibility, EGFP- and HA-tagged WT-Prrt2 and the R308C mutant were transiently transfected to HEK293T cells and Prrt2 expression levels were examined by Western blotting. Significantly reduced protein levels were observed for the R308C mutant compared to WT-Prrt2 (Figure 5), suggesting that this missense mutation may cause disease through reduced expression rather than abnormal trafficking.

### Delayed neuronal migration during development caused by Prrt2 knockdown

**Figure 6:**
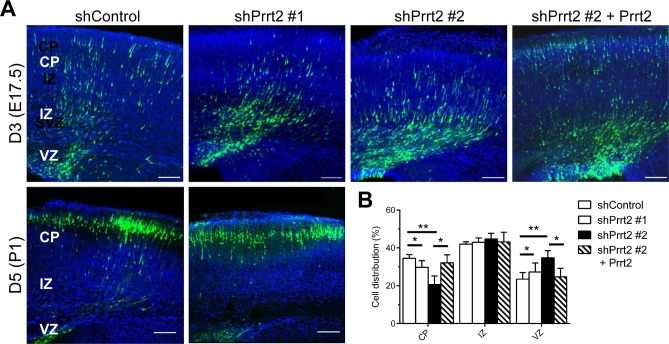
Delay in neuronal migration by Prrt2 knockdown during development **A.** Coronal sections of the mouse brains 3 and 5 days after *in utero* electroporation of shControl or shPrrt2 constructs at E14.5. Three days after electroporation, cells expressing EGFP (green) and shControl had started migration, with many cells having already reached the CP. More cells were restricted to the VZ and IZ in the brains electroporated with each of the shPrrt2. This migration delay was rescued by co-transfection of shPrrt2 and Prrt2 constructs (upper panel). When brain sections were examined 5 days post electroporation, most cells in both shControl and shPrrt2 groups had reached the CP (lower panel). All sections were stained with DAPI (blue) to show the cell nuclei. Scale bars = 100 μm. **B.** Statistical analysis showed significant differences in cell distribution in the CP, IZ and VZ at E17.5for the four conditions. Error bars represent SEM; *: *p* < 0.05, ** : *p* < 0.01.

To explore the loss-of-function effects of Prrt2 in the brain, we transfected Prrt2 shRNA constructs with cDNA expressing EGFP into mouse brains by *in utero* electroporation at embryonic (E) day 14.5 [23, 24]. Using this method, plasmids can be introduced into a subset of neural stem cells (*i.e.* radial glia) during cortical development. The development of electroporated mouse brains was examined 3 and 5 days post electroporation, at E17.5 and postnatal (P) day 1, respectively. Normally, neurons generated from neural stem cells in the ventricular zone (VZ) migrate along radial fibers towards the cortical plate (CP) [25–27]. The neurons in the brains electroporated with Prrt2 shRNA exhibited a delay in neuronal migration from the VZ to the CP, with many cells accumulated at the VZ, subventricular zone (SVZ) and intermediate zone (IZ) (Figure 6A, top panels). Quantification of the cells at day 3 (E17.5) revealed significantly reduced numbers of neurons in the CP, as most of the cells remained in the VZ (Figure 6B). This migration delay was partially rescued by expressing Prrt2 in knockdown cells. Later, at day 5 (P1), most of the neurons in both the control and shRNA-electroporated brains reached the CP (Figure 6A, bottom panels), indicating that neuronal migration was delayed rather than completely blocked during cortical development.

### Decreased dendritic spine density caused by Prrt2 knockdown

**Figure 7:**
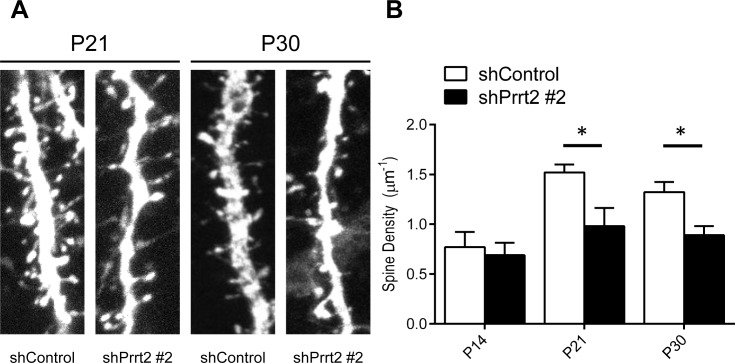
Decreased dendritic spine density after Prrt2 knockdown **A.** Dendritic spines were imaged at P21 and P30 in brains electroporated *in utero* with control or Prrt2 shRNA along with EGFP constructs at E14.5. Lower spine densities for Prrt2 shRNA-expressing pyramidal neurons were observed on representative dendrites at both P21 and P30. **B.** Statistical analysis of spine density of dendrites at P14, P21 and P30. There was no significant difference between control and Prrt2 shRNA-expressing groups at P14. Significant decreases in spine density for Prrt2 shRNA-expressing cells were observed at P21 and P30. *: *p* < 0.05.

Since we documented the presence of Prrt2 at both the pre- and post-synaptic membranes, we hypothesized that loss of normal PRRT2 functions may cause changes in synaptic connections, which could be visualized by the density of dendritic spines [28]. Therefore, we investigated changes in dendritic spines in Prrt2 knockdown mice. Plasmids expressing Prrt2 shRNA and EGFP were electroporated into the developing cortex of mouse embryos *in utero* at E14.5. The mice were allowed to give birth and the brains of their pups were examined at P14, P21 and P30. Pyramidal neurons of layer 2/3 (L2/3) were observed under a confocal or two-photon microscope. We counted the numbers of dendritic spines along apical dendrites (Figure 7A) [29]. At P14, neurons expressing Prrt2 shRNA showed a dendritic spine density similar to neurons in the control brains. However, there were significant decreases in spine density for neurons expressing shPrrt2 #2 at P21 and P30 (Figure 7B). The reduction of dendritic spine density was not observed in the neurons expression shPrrt2 #1 (data not shown). As shPrrt2 #2 exhibited a much higher knockdown efficiency than shPrrt2 #1 (Figure 2C), our results suggested that nearly complete knockdown of Prrt2 may be required for the defect in dendritic spine during neural development and the loss-of-functional effect may be dose-dependent.

## DISCUSSION

In this study, we identified important characteristics of the PRRT2 protein and its potential functions during neuronal and synaptic development. Using our highly specific antibody, we documented the localization of endogenous Prrt2 at both the pre- and post-synaptic membranes. *PRRT2* mutations could lead to abnormal intracellular trafficking or reduced expression. More significantly, our *in vivo* functional assays using Prrt2 shRNAs showed that a nearly complete Prrt2 knockdown resulted in a delay in neuronal migration during early neural development and also defective spinogenesis. These results pointed to the essential functions of PRRT2 during neural development, which were not recognized before this study but may be critical in the pathomechanisms of *PRRT2*-related disorders.

Recent studies have revealed that PRRT2 is a component of the AMPAR (α-amino-3-hydroxy-5-methyl-4-isoxazolepropionic acid receptor) complex [30, 31] and may be involved in neurotransmitter release and glutamate signaling[20, 32]. Consistently, PRRT2 was found to co-localize with pre- and post-synaptic markers vGlut1 and PSD95 in our study. In addition, our study revealed that PRRT2 co-fractionated with SNAP25 and was localized close to it at synapses, supporting the interactions between these two proteins.

We observed a reduced protein level in the HEK293T cells expressing the R308C mutant. Reduced expression has been reported in cells expressing other *PRRT2* mutations [19, 33, 34] and could be a common functional loss of PKD-related mutations. It remains unclear whether the decreased protein level in cells was caused by decreased production, protein instability, or both. Either way, this result suggested that haploinsufficiency resulting from some forms of *PRRT2* mutations may be the pathomechanism of PKD-related disorders. Another loss-of-function effect underlying PKD and related diseases was seen in the truncated forms of Prrt2, which were retained in the cytoplasm with some entering the nucleus.

Using *in utero* electroporation, we found that a further decrease in PRRT2 expression in cortical neurons resulted in delayed neuronal migration in the early stage of development. Furthermore, nearly complete Prrt2 knockdown caused a reduction in dendritic spine density. Consistent with this result, a recent *in vitro* study also showed a decrease in synapses in cultured hippocampal neurons when Prrt2 was knocked down [32]. Delayed neuronal migration and abnormal spine density are frequently associated with various cognitive, learning and memory deficits [28, 34–36]. Our results indicated that these defects may contribute to the severe encephalopathy caused by the rare homozygous or biallelic *PRRT2* mutations [10, 15, 16, 37].

**Figure 8:**
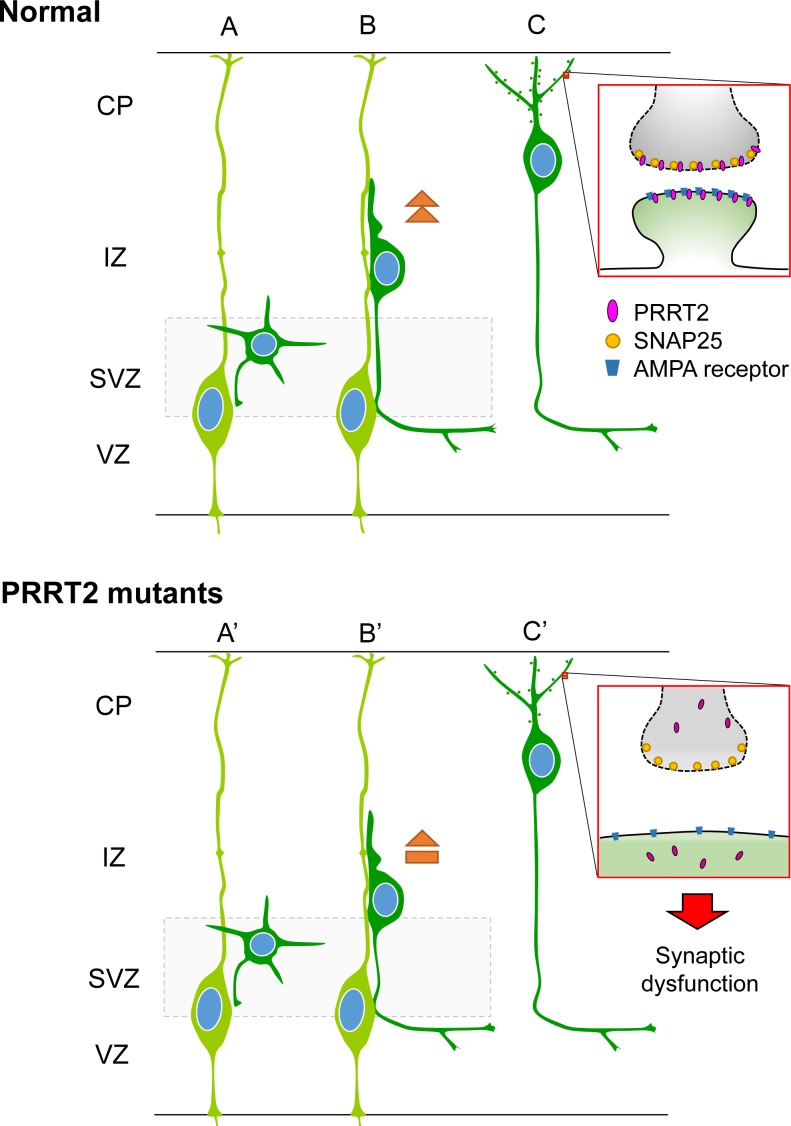
Schematic diagram showing effects of PRRT2 mutation on neuronal migration and dendritic spines in cortical neurons **A.** Radial glia cells (light green) produce postmitotic neurons (dark green). **B.** Postmitotic neurons then migrate along the radial fiber to the CP. **C.** After reaching the cortical plate, the cells extend dendrites and form synaptic connections with other neurons through dendritic spines. Box shows a magnification of the pre- and post-synaptic regions. PRRT2 (pink) is localized at both the pre- and post-synaptic membranes through interactions with SNAP25^18, 25, 44^ and the AMPA receptor^25, 33, 34^. (A', B') In neurons carrying PKD-associated *PRRT2* mutations, reduced expression, mislocalization and other potential defects delay the neuronal migration. (C') PRRT2 mutation also decreases the dendritic spine density and causes synaptic dysfunction, possibly through SNAP25 and/or AMPA receptor pathways.

Together, we proposed the potential mechanism that may underlie various *PRRT2*-related diseases (Figure 8). PRRT2 participates in normal synaptic functions through interactions with SNAP25 [18, 19, 32] and probably other molecules of the AMPAR complex [30, 31]. *PRRT2* mutations cause reduced expression and/or intracellular mislocalization of PRRT2 proteins, leading to their functional loss. PRRT2 loss-of-function can also lead to a delay in neuronal migration and synaptic loss. The diverse pathological impacts of *PRRT2* mutations in turn contribute to the wide phenotypic spectrum, from PKD by heterozygous mutations to severe congenital encephalopathy by homozygous or biallelic mutations.

## MATERIALS AND METHODS

### Antibody production

The Prrt2 antibody was generated using antigen prepared by Nickel resin purification from a bacterially-expressed peptide comprising the extracellular domain (amino acids 1-268) of Prrt2 protein with a 6X His tag. Prrt2 proteins from adult rat brain lysates were detected using the first bleed of Prrt2 anti-serum obtained from a rabbit, whereas the preimmune serum was drawn from the rabbit before antigen injection as the control.

### Cell culture and transient transfection

Cultured hippocampal and cortical neurons were isolated from P0 Sprague-Dawley rats and E18 ICR mice, respectively, according to our previous report [38]. COS-7 (monkey kidney epithelial cells) and HEK293T (human embryonic kidney cells) cell lines were cultured in Dulbecco's Modified Eagle Medium (DMEM, Life Technologies, USA) supplemented with 10% fetal bovine serum. Transfection was carried out using the Lipofectamine 2000 and 3000 lipofection reagents (Life Technologies, USA) and cells were harvested 24-48 hours after transfection.

### Plasmids and antibodies

The mouse *Prrt2* cDNA construct was purchased from Origene (MG214094; GenBank Accession No.: NM_01102563). The enhanced green fluorescent protein-tagged Prrt2 (EGFP-Prrt2) constructs of the wild-type *Prrt2* and the 7 PKD-associated mutations were generated and cloned into the pEGFP-C1 vector (Clontech, Palo Alto, CA). The human influenza hemagglutinin-tagged Prrt2 (HA-Prrt2) constructs were cloned into the pcDNA3.1 (−) vector (Clontech, Palo Alto, CA). All mutant constructs were fully sequenced to verify the mutated nucleotides.

### Western blot analysis

EGFP-Prrt2 and HA-Prrt2 constructs with either wild-type or the point mutation, R308C, were transiently transfected into an HEK293 cell line. Total proteins were harvested at 24 hours after transfection. The protein concentrations were determined using a BCA assay (Thermo Fisher Scientific, USA). The results were quantified using ImageJ software (National Institutes of Health, NIH, USA).

### Knock-down constructs

For RNAi, short hairpin RNA (shRNA) constructs were based on the pLKO.1-puro vector (RNAi core of Academia Sinica, Taiwan) and pGPU6/GFP/Neo vector (Tools, Taiwan). The *Prrt2* target sequences (shPrrt2 #1: 5′-CATCAACTTAGGCGTGTATAA-3′ and shPrrt2 #2: 5′-GGCCACAGACCTCAGTTTAAA-3′) were chosen from a portion of the *Prrt2* coding region, where the sequence was conserved between human and mouse. The empty vector or a scramble (5′-TTCTCCGAACGTGTCACGT-3′) sequence (shControl) was used as the control.

### Immunofluorescence

Cultured mice neurons were fixed in 4% paraformaldehyde/4% sucrose/ phosphate-buffered saline (PBS) solution for 20 minutes at 4°C, followed by PBS rinsing and 0.1% Triton X-100 permeabilization at 4°C for 15 minutes. Neurons were then incubated at room temperature (RT) for 20 minutes in blocking solution (2% BSA/4% normal goat serum/PBS). The primary antibodies were diluted in blocking solution and added to neurons for incubation at 4°C overnight in a humidified chamber, followed by secondary antibody incubation in the dark at RT for 1 hour. Coverslips were mounted in Prolonged Gold Antifade reagent (Life Technologies, USA) and stored at 4°C until imaging.

### Image acquisition

HEK293T cells expressing wild-type or mutated Prrt2 proteins were imaged by a confocal microscope (Zeiss LSM700, Germany) with a 100X Plan-Apochromat NA = 1.4 oil objective. Final composite images were processed by the ImageJ software (NIH, USA).

### Membrane fractionation and post-synaptic density (PSD) purification

The adult rat brain was dissected out for subcellular fractionation as previously described [39]. PSD preparation was performed as described previously [40].

### *In utero* electroporation

Plasmids were transfected using intraventricular injection of embryonic ICR mice *in utero,* followed by electroporation as previously described [26]. Embryos were then perfused transcardially using cold PBS (pH 7.4) followed by 4% paraformaldehyde in PBS. Brains were collected and further postfixed in 4% paraformaldehyde overnight at 4°C. For brain slice preparation, brains were embedded in 4% low-melting-point agarose (Amresco, USA) dissolved in PBS and sectioned using a Vibratome (Leica, Germany) at a thickness of 100 μm.

### Dendritic spine analysis

Mouse brains electroporated with EGFP were fixed, sectioned and imaged with a confocal or two-photon microscope equipped with a 40X objective lens (Zeiss, Germany) as previously described [29]. Apical dendrites extending from the layer (L) 2/3 pyramidal neurons expressing GFP were randomly selected and analyzed semi-automatically with a customized Matlab program. All image analysis and quantification was conducted in a double-blind manner.

Animals were maintained according to protocols approved by the Institutional Animal Care and Use Committee (IACUC) at National Yang-Ming University. All animal studies were conducted in accordance with the United States Public Health Service's Policy on Humane Care and Use of Laboratory Animals.

## Abbreviations

BFIE: benign familial infantile epilepsy
CP: cortical plate
DIV: days *in vitro*
E: embryonic
EA: episodic ataxia
EGFP: enhanced green fluorescent protein
HA: human influenza hemagglutinin
HM: hemiplegic migraine
ICCA: infantile convulsions and choreoathetosis
IZ: intermediate zone
PKD: paroxysmal kinesigenic dyskinesia
P: postnatal
*PRRT2*: the proline-rich transmembrane protein 2 gene
PRRT2: the proline-rich transmembrane protein
PSD: postsynaptic density
RNAi: RNA interference
shRNA: short hairpin RNA
SNAP25: the synaptosomal-associated protein, 25kD
SVZ: subventricular zone
VZ: ventricular zone
